# Few-Flakes Reduced Graphene Oxide Sensors for Organic Vapors with a High Signal-to-Noise Ratio

**DOI:** 10.3390/nano7100339

**Published:** 2017-10-21

**Authors:** Nowzesh Hasan, Wenli Zhang, Adarsh D. Radadia

**Affiliations:** 1 Institute for Micromanufacturing, Center for Biomedical Engineering and Rehabilitation Services, Louisiana Tech University, Ruston, LA 71272, USA; azh002@latech.edu; 2 School of Biomedical Engineering, Fourth Military Medical School, Xi’an 710032, China; wenlizhang1121@163.com

**Keywords:** graphene oxide, reduced graphene oxide, graphene gas sensor

## Abstract

This paper reports our findings on how to prepare a graphene oxide-based gas sensor for sensing fast pulses of volatile organic compounds with a better signal-to-noise ratio. We use rapid acetone pulses of varying concentrations to test the sensors. First, we compare the effect of graphene oxide deposition method (dielectrophoresis versus solvent evaporation) on the sensor’s response. We find that dielectrophoresis yields films with uniform coverage and better sensor response. Second, we examine the effect of chemical reduction. Contrary to prior reports, we find that graphene oxide reduction leads to a reduction in sensor response and current noise, thus keeping the signal-to-noise ratio the same. We found that if we sonicated the sensor in acetone, we created a sensor with a few flakes of reduced graphene oxide. Such sensors provided a higher signal-to-noise ratio that could be correlated to the vapor concentration of acetone with better repeatability. Modeling shows that the sensor’s response is due to one-site Langmuir adsorption or an overall single exponent process. Further, the desorption of acetone as deduced from the sensor recovery signal follows a single exponent process. Thus, we show a simple way to improve the signal-to-noise ratio in reduced graphene oxide sensors.

## 1. Introduction

Nanomaterials, such as graphene [1,2,3,4,5], carbon nanotubes [6,7], nanowires [8,9], and transition metal dichalcogenides [10,11], due to their outstanding electrical and chemical properties, have received great attention to build gas sensors with high selectivity, repeatability, and signal-to-noise ratio (SNR). Of all of these, graphene oxide (GO) and its reduced form (rGO) have received great attention due to their relatively low cost and ease of manufacturing. GO, which has been primarily reported to be prepared by Hummer’s method [12], or its variations, consists of a disrupted sp^2^-hybridized network unlike graphene, and thus it is electrically insulating. Many reduction processes, including chemical [13,14,15,16], thermal [17], and electrochemical [18] processes, have been demonstrated to partly recover the hexagonal sp^2^ network in GO films by removing oxygenated functional groups [19,20]. It is believed that the doping of the graphene plane with the gas molecules induces a change in the resistivity of the sensor. Robinson et al. have demonstrated that reduction of spun coat GO (0.5–3 mg/mL water) using hydrazine hydrate vapor (100 °C) for a longer time (24 h) is the key to improving sensitivity (% change in conductance) to acetone vapor injections (250 ppm, 5 s) [21]. The detection of dinitrotoluene, 2-chloroethylethylsulfide, and dimethymethylphosphonate in parts per billion (ppb) was shown to be feasible. Similarly, Lu et al. have shown that a thermal reduction (200 °C, 1 h) of drop-casted GO solution (0.3 mg/mL) induces a higher detection sensitivity to ammonia and nitrogen dioxide [22]. A subsequent study by them reports that a higher level of reduction can be achieved by the chemical method (hydrazine mono-hydrate in solution phase for 12 h at 80 °C), which thus improves the detection sensitivity to ammonia and nitrogen dioxide [22]. Dua et al. have also shown that a mild and greener process, such as the ascorbic acid-mediated reduction of GO (80 °C, 1 h), results in sensors with similar electrical properties to those obtained via the hydrazine reduction method [23]. The detection of nitrogen dioxide and chlorine gas was demonstrated from 500 ppb to 100 ppm using inkjet-printed GO sensors on poly-ethylene terephthalate substrates using a solvent evaporation process (dynamic vacuum, 60 °C, 12 h). Unlike the findings above, we observe that the reduction of GO reduces the noise but also reduces the signal to fast pulses (2 s) of acetone vapor injections, leading to no enhancement in signal-to-noise ratio (SNR). We find that a solvent-mediated exfoliation of the reduced films is important to improve the SNR.

Further, drop-casting (solvent-evaporation), spin coating, and dielectrophoresis (DEP) have been prevalent methods for preparing GO-based gas sensors [24,25,26,27]. Li et al. [28] have shown that DEP (10 V_p-p_, 10 kHz) results in ordered conductive channels between electrodes in comparison to GO deposition via solvent evaporation (25 °C, 1 atm). Wang et al. have demonstrated an optimization of DEP voltage, frequency, and process time to fabricate a highly sensitive hydrogen gas (200 ppm) sensor [29]. In this paper, we also show that DEP is a better choice for preparing volatile organic vapor sensors.

## 2. Results and Discussion

### 2.1. Impact of Dielectrophoretic Deposition of GO

**Figure 1:**
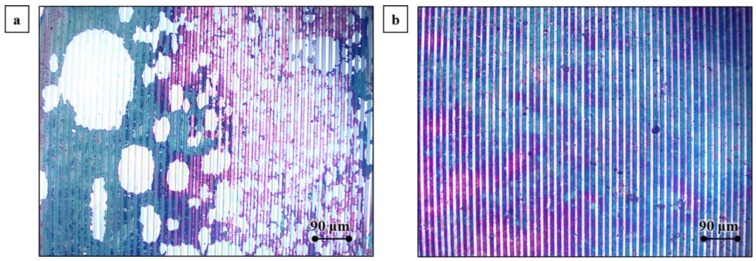

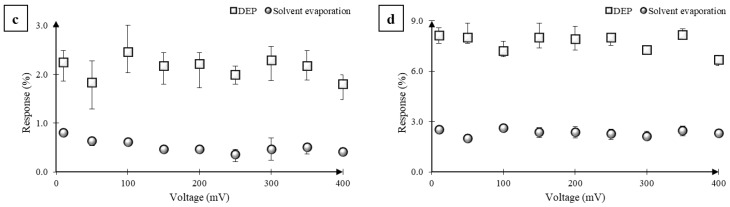
Optical microscope images of graphene oxide (GO) films deposited via (**a**) the solvent evaporation method; and (**b**) the DEP method. Response (%) is the percentage variation of the electrical resistance from GO-coated sensors as a function of direct current (DC) voltage (V) when exposed to 2 s acetone vapor (partial pressure, *P/Po* = 0.2, 25 °C, 1 atm) pulses in two independent experiments (**c**,**d**). The error bars represent the maximum and minimum values of Response (%) obtained from five vapor pulses.

First we compared the GO sensors obtained via solvent evaporation (drop casting) and sensors coated with DEP in terms of their morphology and their sensing performance. The sample microscope images shown in Figure 1a,b show that the solvent evaporation method results in a discontinuous and random deposition, while films deposited with DEP look continuous and uniform. In our past experience, we have found a significant variation in microstructure and hence the electrical property of interdigitated electrode (IDE) pairs [30,31,32]. To avoid the latter differences and clearly delineate the difference in coatings obtained via solvent evaporation and DEP, an IDE pair was coated with GO via solvent evaporation, Response (%) was recorded to acetone vapor pulses as a percentage variation of the electrical resistance, the GO was completely removed via oxygen plasma (Technics parallel plate RIE, 100 W, 20 sccm O_2_, 1 min) and acetone-isopropanol rinse, new GO film was coated using DEP, and Response (%) was recorded to acetone vapor pulses. Figure 1c,d show the Response (%) recorded for two different IDE pairs. In both experiments, GO-coated with DEP led to a higher Response (%) compared to solvent evaporation. To test if Joule heating altered the latter finding, the bias voltage applied to the sensors during testing was varied from 10 mV to 400 mV. We found that regardless of the bias voltage, sensors coated with DEP produced a far superior response. Thus, GO was coated using DEP for the rest of the study.

### 2.2. Impact of Hydrazine Vapor-Assisted Reduction of GO

**Figure 2:**
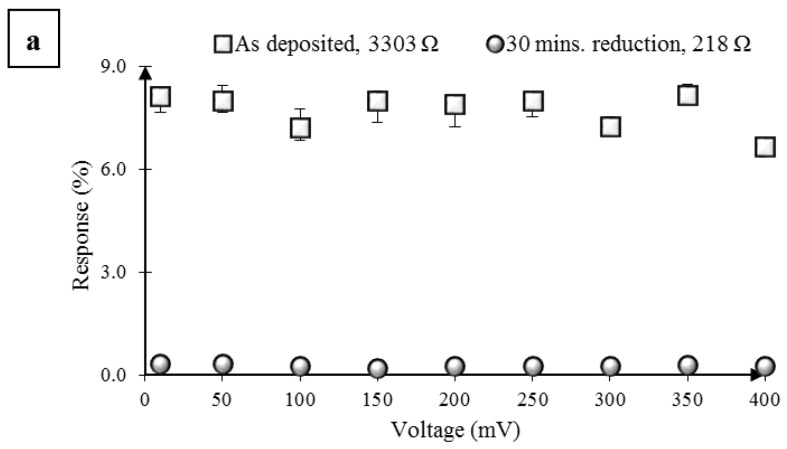

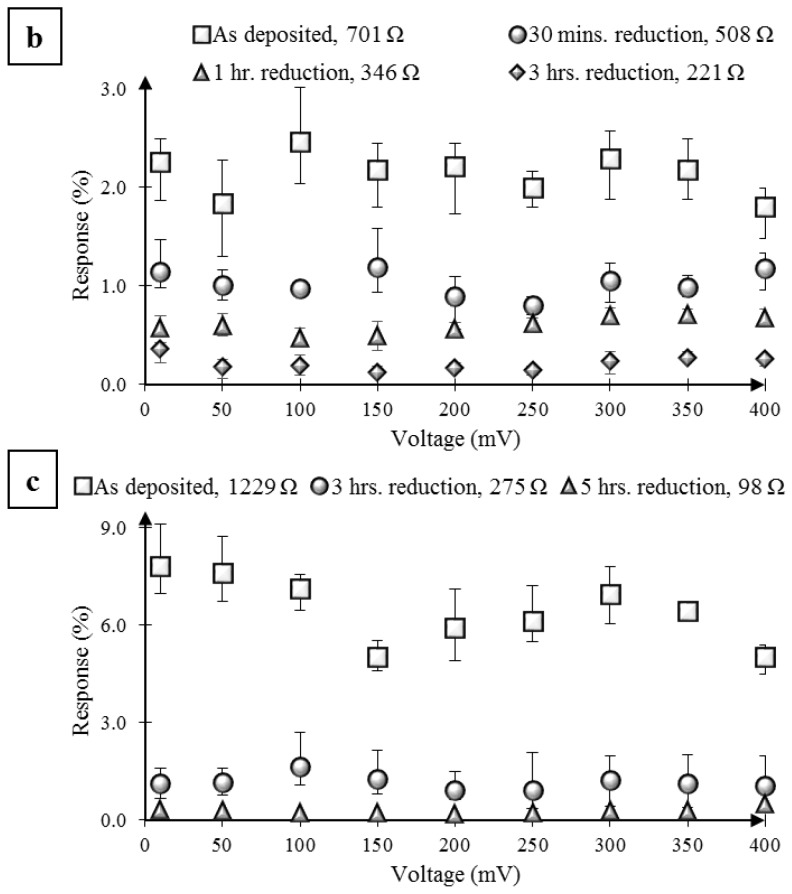
Effect of chemical reduction on a GO sensor’s response to 2 s of acetone vapor pulses (*P/P_o_* = 0.2, 25 °C, 1 atm). Plot of Response (%) versus DC bias for three different GO sensors (**a**–**c**), before and after a hydrazine vapor-assisted reduction for varying times. (**a**) Response (%) from sensor S1 without any reduction (open squares), and after 30 min of reduction (open circles); (**b**) Response (%) from sensor S2 without any reduction (open squares), after 30 min of reduction (open circles), after 1 h of reduction (open triangles), and after 3 h of reduction (open diamonds); (**c**) Response (%) from sensor S3 without reduction (open squares), after 3 h of reduction (open circles), and after 5 h of reduction (open triangles). The error bars represent the maximum and minimum values of Response (%) obtained from five vapor pulses.

**Figure 3:**
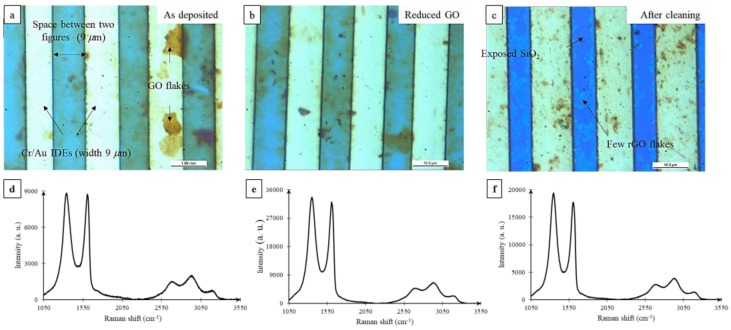
Optical image (**a**) of interdigitated electrodes (IDEs) with as-deposited GO; (**b**) IDEs with reduced graphene oxide (rGO); and (**c**) IDEs with solvent-exfoliated rGO. Raman spectra of (**d**) as-deposited GO; (**e**) rGO; and (**f**) solvent-exfoliated rGO.

Next, we investigated the effect of GO reduction using a hydrazine vapor treatment similar to that reported by Robinson et al. [21]. Three GO sensors (S1, S2, S3) were tested and Response (%) was recorded as a function of bias voltage as shown in Figure 2. The first sensor (S1), with an average resistance of 3303 Ω and without any reduction, showed a response of around 8% to acetone pulses. After 30 min of reduction, the average resistance for the first sensor dropped to 218 Ω, clearly indicating the reduction of GO; however, the response to the acetone pulses dropped to below 1%. Likewise, two more GO sensors (S2 and S3) were tested as shown in Figure 2b,c. On average, about a 23-times lower Response (%) was obtained after 5 h of reduction. The drop in resistance with increased reduction time can be explained by the restoration of the π network in the GO films; however, the drop in the Response (%) with increased reduction can only be explained by the reduced acetone adsorption at electrically active sites. Further, a variation of sensor bias from 10 to 400 mV during testing showed a similar Response (%) and hence a negligible effect of any Joule heating on sensor operation. The effect of reduction on gas sensor Response (%) obtained from our experiments agree with those reported by Prezioso et al. [33]. but are contrary to those reported prior by Robinson et al., Dua et al., and Lu et al. Optical microscopy studies of the GO films pre and post reduction indicated no changes in the film morphology as shown in Figure 3a,b. The rGO sensors were tested post reduction without exposure to a solvent wash. Next, we subjected the rGO sensor S3 from Figure 2c to sonication in acetone for 5 min, followed by a quick rinse with isopropyl alcohol and deionized water (DI), and drying under a gentle stream of nitrogen. We found that we could remove the majority of the rGO flakes, leaving behind a few thin flakes abridging the IDE pairs as shown in Figure 3c.

**Figure 4:**
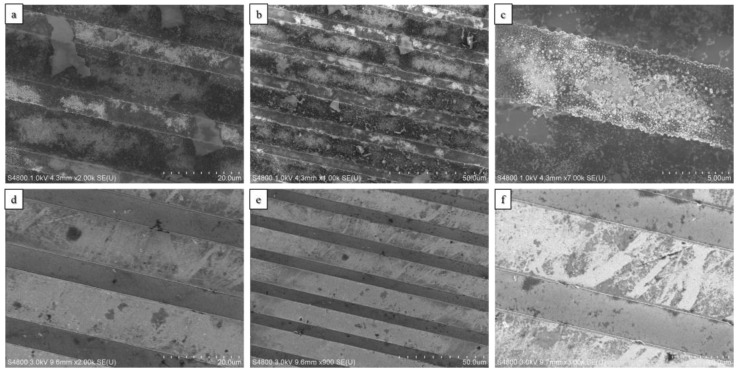
Scanning electron microscope (SEM) image (**a**–**c**) of IDEs with as-deposited GO; and (**d**–**f**) IDEs with solvent-exfoliated rGO.

**Figure 5:**
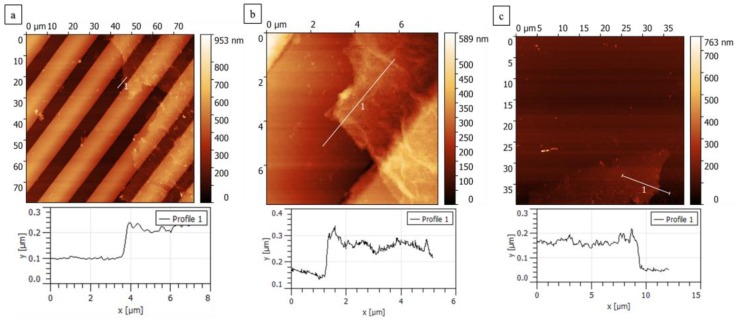
Atomic force microscopy images of few-flakes GO on (**a**) the IDE; (**b**) between two electrodes; and (**c**) on a flat piece of silicon. The label “1” indicates the sectioning line along with the height profile that was obtained as shown below each image.

We studied the structural changes for the as-deposited GO, rGO, and the few-flakes rGO using Raman spectroscopy as shown in Figure 3d–f. The peak analysis is presented in Supplementary Material Table S1. The Raman spectrum of the films was characterized by the first-order region (up to 2000 cm^−1^) fitted by two Lorentzian curves: the G-band observed at 1606 cm^−1^ and the D-band at 1341.9 cm^−1^. The second-order Raman peaks were fitted to three Lorentzian curves: the 2D-band observed at 2682.2 cm^−1^, the S3 or (D + G) band at 2959.9 cm^−1^, and the C–H mode stretching band at 3186.6 cm^−1^. The G-band corresponds to the high frequency first-order scattering of E_2*g*_ phonon of sp^2^ carbon atoms [16,34]. The D-band peak is due to the breathing modes of six atoms rings [34], which is an indication of disorder emerging from defects such as vacancies, grain boundaries, and amorphous carbon species [35]. The 2D-band is the D-band overtone, and the 2D band comes where momentum conservation is satisfied by two phonons with opposite wave vectors. The S3 band is the second order peak derived from the “one phonon” peaks of the bands D and G [36]. The reduction of graphene oxide films has been reported by Moon et al. and Stankovich et al. to increase the intensity ratio, *I_D_/I_G_* [16,37]. Using Raman characterization (spot size 2.6 μm, 532 nm, 8.5 mW at sample), we found a slight increase in band intensity ratio, *I_D_/I_G_*, from 1.01 for the as-deposited GO to 1.041 after the hydrazine vapor reduction, and 1.079 after the solvent exfoliation process. Due to reduction and solvent exfoliation, the peak intensity of the D-band was found to increase from 8442.5 to 32,495.9 upon reduction and reduce to 18,865.5 upon solvent exfoliation. Similarly, the G-band intensity was found to increase from 8382.5 to 31,215.9 upon reduction, and then decrease to 17,481.5 upon solvent exfoliation. The increment in intensities of the first-order scattering peaks (D-band and G-band) indicates a better graphitization by decreasing the average size of the sp^2^ domain through the reduction process, which lowers the oxygen content as well [38]. Upon solvent exfoliation of the rGO, the D and the 2D peaks were found to shift to higher wavenumbers, while the full width at half maximum was found to reduce. This indicates that solvent exfoliation resulted in a thinner film than before [39]. Scanning electron microscopy (Figure 4) and atomic force microscopy (Figure 5) of these films verify the relatively thick nature of the as-deposited GO. However, upon exfoliation, the few-flakes rGO is only 100–200 nm thick.

### 2.3. Impact of Solvent-Assisted Exfoliation of Reduced GO

**Figure 6:**
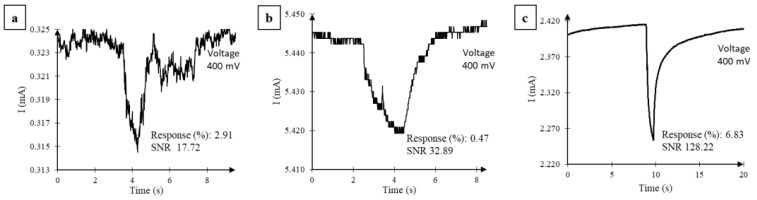

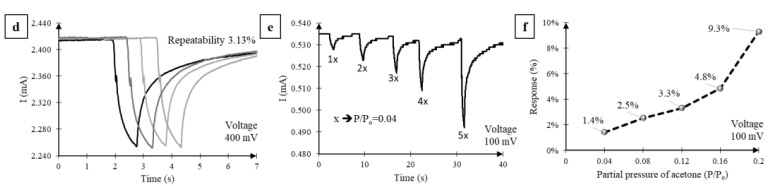
Sensor current in response to 2 s of acetone vapor (*P/P_o_* = 0.2, 25 °C, 1 atm) pulses observed on GO sensor S3 (**a**) as-deposited; (**b**) after 5 h of reduction with hydrazine vapor; and (**c**) after solvent exfoliation. DC bias was held constant at 400 mV. (**d**) Current responses to four acetone vapor pulses obtained from the solvent-exfoliated rGO sensor (different colored lines represent different trials). (**e**) Current response to 2 s of acetone vapor pulses of varying partial pressure from the solvent-exfoliated rGO sensor. (**f**) Response (%) calculated for signals in (**e**) plotted versus the partial pressure of acetone used to test response. SNR: signal-to-noise ratio.

The sensors with few flakes obtained after solvent exfoliation demonstrated a sharp response to acetone injections as shown in Figure 6c, when compared to the as-deposited (Figure 6a) and reduced (Figure 6b) states of the same sensor. For a bias range of 10 mV to 400 mV, an average response was found to be 6.83%, which is more than twice that obtained before reduction (2.91%) or after reduction (0.47%); however, the average resistance of the sensor due to sonication in acetone was found to only increase from 98 to 184 Ω, which is significantly lower resistance than that of the as-deposited GO film (1229 Ω). This indicates that the flakes that remain after solvent exfoliation are in a reduced state. Most importantly, the noise of the sensor was significantly lower, thus resulting in a sharp signal. Repeatability, which is the ability of a sensor to represent the same value under identical conditions, was calculated as the standard deviation in maximum current change observed (*I*_signal_). We found that solvent treatment of the rGO sensor induced better repeatability, ~3.13% as shown Figure 6d. Further, when the partial pressure of acetone was varied from 0.04 to 0.2, as shown in Figure 6e, the Response (%) as analyzed in Figure 6f was found to be a linear function of the acetone’s partial pressure from 0.04 to 0.16, suggesting a sensitivity of 0.29 Response (%) per partial pressure fraction. Assuming the smallest signal can be measured with an SNR of 3, the lower limit of detection for acetone would be a *P/P_o_* = 0.018 (25 °C, 1 atm).

**Figure 7:**
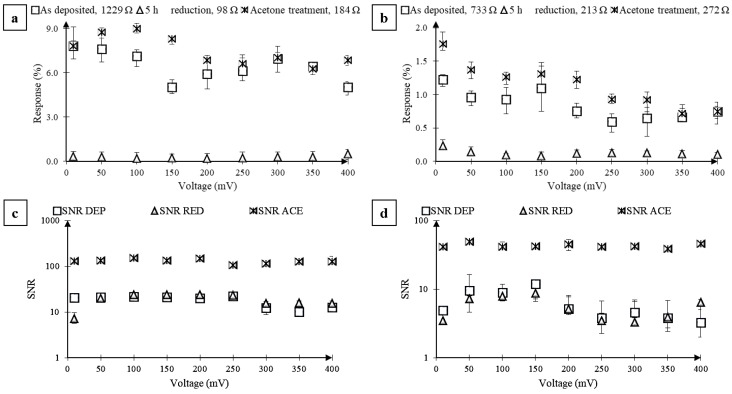
Effect of solvent-mediated cleaning on the response of GO sensors S3 and S4 to 2 s of acetone vapor pulses (*P/P_o_* = 0.2, 25 °C, 1 atm). Response (%) (**a**,**b**) and SNR (**c**,**d**) from two different sensors with as-deposited GO film, after 5 h of reduction with hydrazine vapor, and solvent-mediated cleaning of the reduced GO film. (**a**) Response (%) from first sensor without any reduction (open squares), after 5 h of reduction (open triangles), and after cleaning (asterisks). (**b**) Response (%) from the second sensor without any reduction (open squares), after 5 h of reduction (open triangles), and after cleaning (asterisks). (**c**) SNR from the first sensor without any reduction (open squares), after 5 h of reduction (open triangles), and after cleaning (asterisks). (**d**) SNR from the second sensor without any reduction (open squares), after 5 h of reduction (open triangles), and after cleaning (asterisks). The error bars represent the maximum and minimum values of the Response (%) and SNR obtained from five vapor pulses. Some error bars for the SNR are difficult to see on the semi-log plot.

Figure 7 shows the characterization of two different sensors with respect to their as-deposited state and the reduced state: Figure 7a,c for sensor S3; and Figure 7b,d for sensor S4. As shown in Figure 7a, within the bias range of 10 to 400 mV, solvent exfoliation of the reduced sensor S3 helped recover the sensor response that was diminished by hydrazine reduction. Replicability of these findings was verified using a new sensor S4 that was fabricated and tested in an identical manner. As shown in Figure 7b for S4, within the bias range of 10 to 400 mV, the average Response (1.14%) to acetone pulses was found to be similar or more compared to that obtained before reduction (0.85%) but nearly an order magnitude higher than that obtained after 5 h of reduction (0.13%); however, the average resistance of the sensor was found to only increase from 213 to 272 Ω, which is significantly lower than that before reduction (733 Ω). Also, similar to sensor S3, we found that the solvent exfoliation of S4 resulted in a significant reduction in noise. Figure 7c,d shows the SNR calculated for sensors S3 and S4 in its different states: as-deposited, reduced, and solvent-exfoliated. The lower SNR after the hydrazine reduction of as-deposited GO can be explained by the lower density of states. The average SNR for the rGO sensor after exfoliation increased by an order magnitude (128.22) when compared to before reduction (17.72) or after reduction (32.89). On sensor S4, the SNR after GO deposition and after reduction was 6.22 and 5.54, respectively, while solvent exfoliation increased the SNR to 42.98, which is more than an order of magnitude.

The adsorption of acetone during testing is hypothesized to occur only at the exposed planes of the rGO. The charge carrier transport through the bulk of the rGO was thus not affected by exposure to acetone vapor. The sonication in the solvent led to an exfoliation of the multi-layer rGO, leaving behind a significantly thinner film with electrical characteristics that could then be altered significantly by exposure to acetone vapor. To further delineate the differences in the physisorption kinetics of acetone vapor on GO, rGO, or few-flakes rGO, we fit the sensor response to different models.

### 2.4. Fitting Data to Langmuir Adsorption Models

The physisorption of acetone on a GO or reduced GO surface during sensor response can be modeled using the Langmuir one-site or two-site model. First, the fractional occupancy of the adsorption sites (*θ*) on a GO or rGO surface was calculated as:

(1) $$\theta (t)=\frac{I_{\max}-I(t)}{I_{\max}-I_{\min}},$$

where *I*_max_ and *I*_min_ are the electrical current responses while the fractional occupancy is zero and 100% respectively, and *I(t)* represents any particular current value at time *t*. The fractional occupancy of the adsorption sites (*θ*) in the one-site Langmuir isotherm model can be expressed as [40]:

(2) $$\theta (t)=\frac{1-e^{\left(-\alpha \beta \;t\right)}}{\alpha },$$

where $\alpha =1+\frac{1}{KC}$, $\beta =\frac{k_{a}C}{N_{\circ }}$, and $K=\frac{k_{a}}{k_{d}}$, where *C* is the concentration of the adsorbate, *k_a_* and *k_d_* are the rate of adsorption and desorption, respectively, and *N*_o_ is the surface adsorbate concentration at full coverage. The fractional occupancy of the adsorption sites (*θ*) in the two-site Langmuir isotherm model can be expressed as:

(3) $$\theta (t)=\frac{1-e^{\delta \;t}}{\left(\alpha -\frac{e^{\delta \;t}}{\alpha }\right)},$$

(4) $$\delta =\beta (\alpha -\frac{1}{\alpha })=\frac{k_{d}}{N_{\circ }}(1+\frac{k_{a}C}{k_{a}C+1}),$$

As shown in Supplementary Material Figure S1 (A–L), the adsorption on GO, rGO, and few-flakes GO were found to follow the one-site Langmuir model; the parametric values associated with this model are tabulated in Supplementary Material Table S2. When sensors were operated at a 10 mV bias, the one-site Langmuir model parameters were found to be as follows: *α* = 1.29 ± 0.19 and *β* = 4.33 ± 0.63 for GO; *β* = 7.41 ± 11.54 with *α* fixed to 1 for rGO; and *α* = 1.07 ± 0.13 and *β* = 4.13 ± 1.20 for few-flakes rGO. For sensors operated at a 400 mV bias, the one-site Langmuir model parameters were found to be as follows: *α* = 1.28 ± 0.23 and *β* = 4.14 ± 1.26 for GO; *β* = 6.36 ± 1.59 with *α* fixed to 1 for rGO; and *α* = 1.08 ± 0.11 and *β* = 3.67 ± 0.47 for few-flakes rGO. The probability values from a two-tailed *t*-test for *α* values obtained between operation at 10 and 400 mV were 0.96 for GO and 0.91 for few-flakes rGO. Similarly, the probability values from a two-tailed *t*-test for *β* values obtained between operation at 10 and 400 mV were 0.77 for GO, 0.95 for rGO, and 0.47 for few-flakes rGO. This indicates that the operation bias up to 400 mV does not interfere in the adsorption of acetone. Further, we find that the *β* values for GO and few-flakes GO are not statistically different, but they are statistically lower than that for rGO. This indicates that the adsorption rate constant for rGO was higher than that for GO and few-flakes rGO; thus, the data implies that the rGO layer has higher adhesion to acetone. The unshared two pairs of electrons on each adsorbed acetone molecule may further result in increased noise characteristics, such as those observed in our experiments with rGO sensors. The value of α was slightly higher (statistically insignificant) for GO than that for rGO or few-flakes rGO, indicating what may be a comparatively higher desorption rate constant on GO. This indicates that restoring the sp^2^ network of GO may be responsible for the slow desorption of acetone.

### 2.5. Fitting Data to Single Exponent and Double Exponent Models

Prior reports have also characterized sensor responses to single exponent and double exponent models [41]. Likewise, we also modeled acetone adsorption and desorption using the sensor response and recovery signal, respectively, and fit it to both the single and double exponent models. The absolute response of the sensor was calculated as $\frac{I(t)-I_{\circ }}{I_{\circ }}$, where *I*_o_ is the average current value until the sensor starts to respond, and *I(t)* is the current value for any particular time, *t*. For the adsorption mechanism, the single exponent and the double exponent model can be expressed as:

(5) $$\exp{\left(t\right)}_{1}=a\times (1-e^{\frac{t-t_{o}}{\tau }}),$$

(6) $$\exp{\left(t\right)}_{2}=a_{1}\times (1-e^{\frac{t-t_{o1}}{\tau_{1}}})+a_{2}\times (1-e^{\frac{t-t_{o2}}{\tau_{2}}}),$$

where exp(*t*)_1_ and exp(*t*)_2_ represent the single exponent and the double exponent adsorption models, respectively; *a*, *a*_1_, *a*_2_, *t*_o_, *t*_o1_, and *t*_o2_ are constants; and *τ*, *τ*_1_, and *τ*_2_ are the corresponding time constants. Supplementary Material Figure S2 (A–L) shows that the adsorption on GO, few-flakes rGO, and rGO sensors follows the single exponent model. Supplementary Material Table S3 shows that the time constant values were more consistent and the fitting errors for the time constants were lower than that of the double exponent model. The best fit we obtained using the single exponent model for few-flakes rGO (400 mV: *τ* = 0.24 ± 0.02, 10 mV: *τ* = 0.24 ± 0.05). The GO data fit with significant error (400 mV: *τ* = 0.32 ± 0.23, 10 mV: *τ* = 0.16 ± 0.04). The noisy data for rGO made it difficult to fit either exponent models (400 mV: *τ* = 155.29 ± 259.91, 10 mV: *τ* = 347 ± 648). The high error of fit prohibits us from making a comparison of the time constants for adsorption. Also, a direct comparison of the time constants with those reported in the literature is not appropriate because most of the articles report time constants for a longer duration of exposure, while in our case we expose the sensor to 2 s of pulses. Regardless, a time constant of 0.24 ± 0.02 for the few-flakes rGO indicates its potential for application as a gas chromatography detector. Also, we did not see a statistically significant difference in the time constants for the few-flakes rGO sensor operated at 10 and 400 mV, which indicates similar adsorption kinetics at 10 and 400 mV sensor biases and no effect of Joule heating.

The desorption of acetone was also modeled by the recovery part of the signal using the single exponent and the double exponent models as:

(7) $$\exp{\left(t\right)}_{1}=a\times (e^{\frac{t-t_{o}}{\tau }}),$$

(8) $$\exp{\left(t\right)}_{2}=a_{1}\times (e^{\frac{t-t_{o1}}{\tau_{1}}})+a_{2}\times (e^{\frac{t-t_{\circ 2}}{\tau_{2}}}).$$

Supplementary Material Figure S3 (A–L) shows that desorption on the GO, rGO, and few-flakes GO sensors follows the single exponent model. Supplementary Material Table S4 shows that the time constant values were more consistent and the fitting errors for the time constants were lower than that of the double exponent model. The best fit we obtained using the single exponent model for few flakes rGO (400 mV: *τ* = 0.24 ± 0.04, 10 mV: *τ* = 0.36 ± 0.08). The GO data fit with significant error (400 mV: *τ* = 0.66 ± 0.38 after excluding a couple of runs, 10 mV: *τ* = 0.26 ± 0.06). The noisy data for rGO made it difficult to fit either of the exponent models (400 mV: *τ* = 428.31 ± 286.38, 10 mV: *τ* = 280 ± 119.76; one run excluded in each case). We noticed that the time constant for the response and the recovery part of the signals on the few-flakes rGO was similar when operated at 400 mV (0.24 ± 0.04 vs 0.24 ± 0.02); however, the recovery was seen to be slower than the response at 10 mV. This may be attributed to the Joule heating effects of sensor bias.

In summary, our report provides important practical findings in the process of creating GO-based volatile organic compound sensors for pulsed injections, such as those found in gas chromatography. Principally, we show the following. First, a direct comparison of sensing response from GO deposited via DEP and solvent evaporation. Two sensors prepared with DEP on average showed 3–4 times the Response (%) that was demonstrated using solvent evaporation. Second, the impact of chemical reduction using hydrazine hydrate vapors on Response (%) and SNR. Although with an increased duration of chemical reduction the resistance of the three different sensors was seen to decrease, in contrast to prior journal reports, the Response (%) to acetone pulses was found to decrease with an increased duration of chemical reduction, while the SNR remained the same. Third, by sonication exfoliation in acetone, we exfoliate the graphene films leaving behind only a few flakes on the sensor. This few-flakes rGO sensor produces a higher sensor Response (%) (6.83% versus 0.34% without solvent exfoliation) with a higher SNR (130 versus 20 without solvent exfoliation) and good repeatability (Standard deviation in Response (%) was ~3.13%). Further, the Response (%) was quantifiable with respect to acetone vapor pressure. Fourth, the current response and recovery upon exposure to 2 s of acetone pulses followed the single exponent model and not the double exponent model, while the current response part also followed the one-site Langmuir model and not the two-site Langmuir model. This indicates that mostly one type of interaction between the acetone molecules and the rGO lattice was responsible for the current response observed from the short acetone pulses. Although the present results pertain to the detection of acetone, the sensors also showed response to other organic vapors, such as methanol, ethanol, isopropanol, and chloroform. We believe our study introduces an improved way of making GO-based sensors and a further understanding of their operation behavior as enhanced volatile organic compounds sensors uniquely suited for applications in high resolution portable instruments, such as micro gas chromatographs.

## 3. Materials and Methods

### 3.1. Device Fabrication

**Figure 8:**
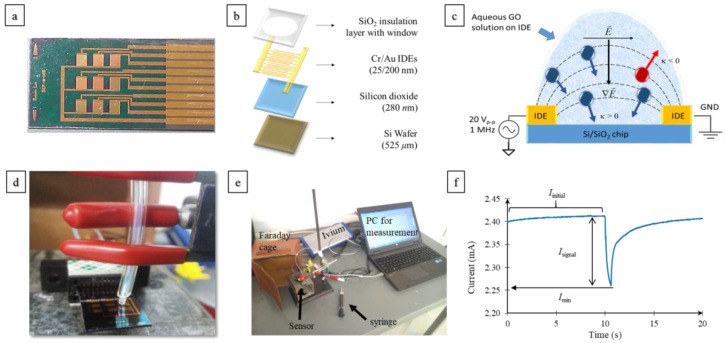
(**a**) Image of the chip with a 3 × 3 array of IDEs. The chip is 2.8 cm in length and 1.4 cm in width. Circular windows of 1.3 mm diameter were opened in the insulator layer over each IDE to expose electrodes for GO deposition. (**b**) Exploded view of the IDE fabricated on a silicon/silicon dioxide substrate consisting of a Cr/Au IDE pair coated with an oxide layer. (**c**) Depiction of the non-uniform electric field applied horizontally during DEP deposition of GO. Blue particles have material properties that result in a Clausius–Mossotti (CM) factor, κ >0, which results in a DEP force directing it towards the chip surface, also known as positive DEP. Red particles have material properties that result in a CM factor, κ <0, which results in a DEP force directing it away from the chip surface, also known as negative DEP. At 1 MHz, the CM factor for GO leads to a positive DEP leading to GO deposition between the IDE fingers. (**d**) Image of the test setup with a gas outlet positioned at a fixed distance over a selected IDE. (**e**) Image of the measurement setup where IDE arrays were electrically connected through a high-density card edge connector to CompactStat^®^ (Ivium Technologies, Eindhoven, the Netherlands) for amperometric detection. The device was set inside a Faraday cage. Chemical vapors were injected using a glass syringe and polytetrafluoroethylene (PTFE) tubing. (**f**) Sample sensor response to acetone vapor injection and graphical illustration of how *I*_signal_, *I*_initial_, and *I*_min_ were measured. PC: personal computer.

The fabrication of devices has been described in detail previously [30,31]. Briefly, devices were fabricated on a silicon wafer (525 ± 25 μm thick, 1–10 Ω·m) with a 280 nm-thick thermal oxide. The IDE layer is composed of a sputter-deposited 25 nm-thick Cr adhesion layer and a 200 nm-thick Au layer, patterned via lift-off as shown in Figure 8a,b. Each chip consisted of a 3 × 3 array of IDE pairs, where each pair consisted of sixty-five fingers, each 2.5 mm long, 9 μm wide, and spaced 9 μm apart. A 300 nm-thick insulating oxide layer was deposited by plasma-enhanced chemical vapor deposition. The IDEs were then exposed by opening circular windows (1.3 mm diameter) in the insulation layer using photolithography and buffered oxide etching. The silicon wafer was then diced to obtain individual devices.

### 3.2. Graphene Oxide Deposition

An aqueous GO dispersion (500 mg/L) with a content ratio of carbon to oxygen of 79%:20% (measured by “5800 ESCA System” X-ray Photoetectron Spectroscopy or XPS (Physical Electronics, Inc., Chanhassen, MN, USA) was produced via Hummer’s method [12]. GO was deposited across IDEs via the solvent evaporation method or the DEP method. GO solution was always mixed for a minute via vortexing prior to use. For solvent evaporation, a 2 μL droplet of GO solution was allowed to evaporate for 10 min at room temperature; the residual solution was blown off the chip with a gentle nitrogen stream, and the chip was baked at 60 °C for 5 min to reduce moisture.

For a simple dielectric homogeneous sphere having radius (*r*) in a medium with permittivity $\varepsilon_{m}$, the DEP force can be expressed as:

(9) F→DEP=2π(r)3εmRe|κ→(ω)|⋅∇E→2,

where *ω* is the angular frequency of the applied field, $\vec{E}$ is the complex applied electric field (as shown in Figure 8c), and (Re|κ→(ω)|) is the real part of the Clausius–Mossotti (CM) factor, $\vec{\kappa }(\omega )$. The latter can be expressed as:

(10) Re(κ→(ω))=εp−εmεp+2εm+3(εmσp−εpσm)τMW(σp+2σm)2(1+ω2τMW2),

where $\varepsilon_{p}$ and $\varepsilon_{m}$ are the real part of permittivity, $\sigma_{p}$ and $\sigma_{m}$ are the conductivity for the dispersed particle and the medium, respectively, and $\tau_{MW}$ is the Maxwell–Wagner charge relaxation time. The latter can be expressed as:

(11) $$\tau_{MW}=\frac{\varepsilon_{p}+\varepsilon_{m}}{\sigma_{p}+2\sigma_{m}}.$$

In the present study, for aqueous dispersed GO solution, the dielectric particles GO (conductivity, $\sigma_{p}$ = 300 S/cm, $\varepsilon_{p}=3.5\varepsilon_{o}$ [42,43]) were suspended in the aqueous medium: water (conductivity, $\sigma_{m}$ = 1 S/cm, $\varepsilon_{m}^{}=80\varepsilon_{o}$ [31,44]), where ($\varepsilon_{o}$) is the permittivity of vacuum. For a DEP frequency lower than 5 × 10^5^ Hz, the second term in Equation (2) plays significant role and leads to a positive CM factor, whereas at a DEP frequency greater than 1 × 10^13^ Hz, the second term in Equation (2) becomes negligible and the CM factor turns negative. For DEP deposition of GO film, a 20 V peak-to-peak, 1 MHz square wave signal was applied for 10 min using a signal generator (Syscomp WGM 201, Toronto, ON, Canada); this setting has been found to be optimal for conformal coatings in prior studies [24,27,42]. Following this, the droplet was blown off the chip using a gentle nitrogen stream and the chip was baked at 60 °C for 5 min to reduce moisture.

### 3.3. Reduction of GO

GO reduction was carried out by placing GO-deposited chips next to a vial containing 50 μL liquid hydrazine hydrate (reagent grade, 50%–60%, M_W_ = 32.05 g/mol, Sigma-Aldrich^®^, St. Louis County, MO, USA) inside a glass jar, which was heated to 100 °C for times varying from 30 min to 5 h.

### 3.4. Sensing Circuit Setup

The GO-sensing microarrays were electrically connected using a high-density card edge connector (Sullins GBB10DHLD, Digi-Key Electronics, MN, USA) to CompactStat^®^ (Ivium Technologies, Eindhoven, the Netherlands) for amperometric detection. The working electrode and the sense electrodes on the CompactStat were short circuited, and the reference lead short circuited to the counter. The vapor injector outlet was held with a holder over a selected sensor as shown in Figure 8d, where a fixed spacing of 1 mm was maintained between the IDE and the vapor outlet for every measurement. Acetone headspace was sampled, diluted as needed, and injected using a 10 mL glass syringe as shown in Figure 8e.

### 3.5. Sensor Signal Characterization

Sensor responses were measured through amperometric detection, where a constant DC voltage (*V*) was applied across an IDE pair over a fixed amount of time, and the corresponding electric current response was measured with a sampling frequency of 100 Hz. The humidity during our testing ranged from 45% to 55% relative humidity (RH) at 20–22 °C. An increase in resistance was observed on exposure to acetone. This is because electrical conduction in graphene in an ambient environment under no gating effect is primarily due to holes, and the lone pair of electrons from the oxygen atom in acetone causes electron–hole recombination, resulting in an increase in resistance [45]. Figure 8f shows a sample response obtained from a sensor and its various aspects used in calculating the Response (%). *I*_initial_ was calculated as the average current until sensor response was discernable (SNR >3). *I*_min_ was calculated as the minimum value current recorded during the response. *I*_signal_ was calculated as (*I*_initial_ − *I*_min_). The amplitude of noise current (*I*_noise_) was defined as the standard deviation among current values recorded for 5 s prior to the signal. SNR was defined as *I*_signal_/*I*_noise_. *R*_initial_ and *R*_response_ were calculated as *V/I*_initial_ and *V/I*_min_. Response (%) was calculated as $\left(\frac{R_{initial}-R_{response}}{R_{initial}}\right)\times 100$.

## Supplementary Materials

The following are available online at http://www.mdpi.com/2079-4991/7/10/339/s1, Figure S1: Fitting sensor response to Langmuir adsorption models, Figure S2: Fitting sensor response to exponent models, Figure S3: Fitting sensor recovery signal to exponent models, Table S1: Peak fit parameters for Raman data, Table S2: Fit parameters for sensor response to Langmuir one-site model, Table S3: Fit parameters for sensor response to exponent models, Table S4: Fit parameters for sensor recovery signal to exponent models.
