# Supplementary material for: Few-Flakes Reduced Graphene Oxide Sensors for Organic Vapors with a High Signal-to-Noise Ratio
Source: Nanomaterials (Basel). 2017 Oct 21;7(10):339. doi: 10.3390/nano7100339 (PMC5666504; doi:10.3390/nano7100339)
Supplement: Supplementary file 1 [file nanomaterials-07-00339-s001.pdf]

Few-Flakes Reduced Graphene Oxide Sensors for Organic Vapors with High Signal-to-Noise Ratio

Nowzesh Hasan[a], Wenli Zhang[a], Adarsh D. Radadia[a, *]

[a]Institute for Micromanufacturing, Center for Biomedical Engineering and Rehabilitation Services, Louisiana Tech University, Ruston, LA 71272, USA.

* Corresponding author. Adarsh D. Radadia, Tel.: +1 318 257 5112. E-mail address: radadia@latech.edu

Supplementary material

Figures:

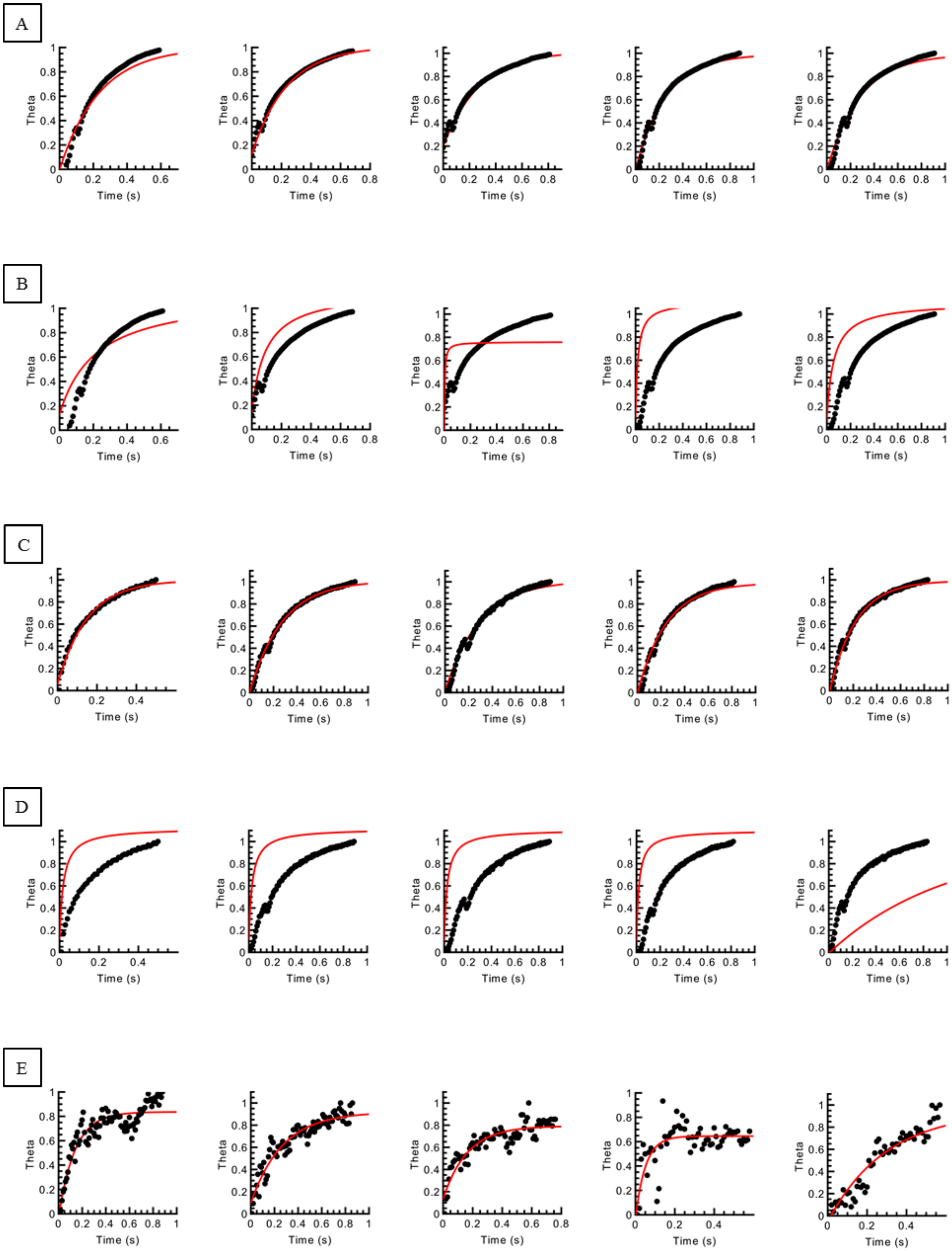
A
B
C
D
E
Theta
Time (s)

F

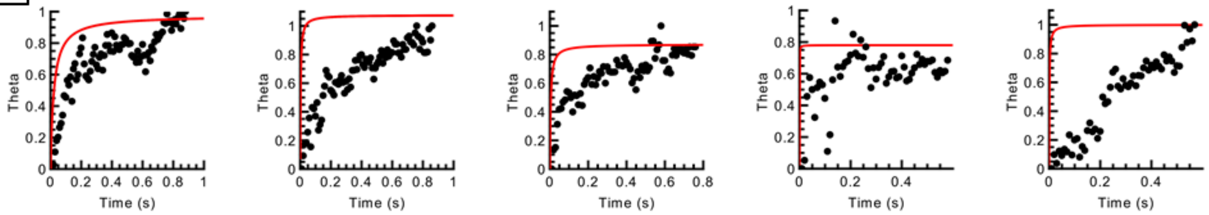
Theta
Time (s)

G

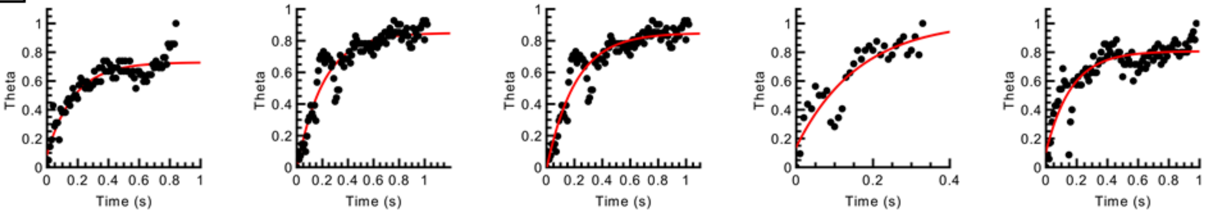
Theta
Time (s)

H

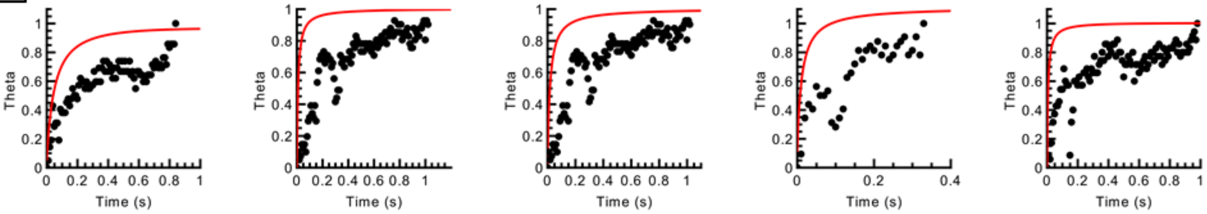
Theta
Time (s)

I

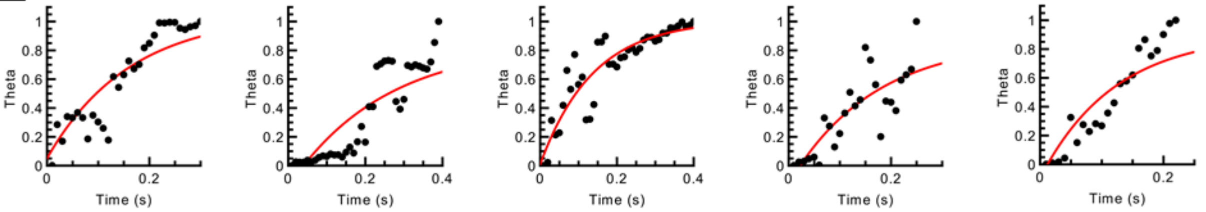
Theta
Time (s)

J

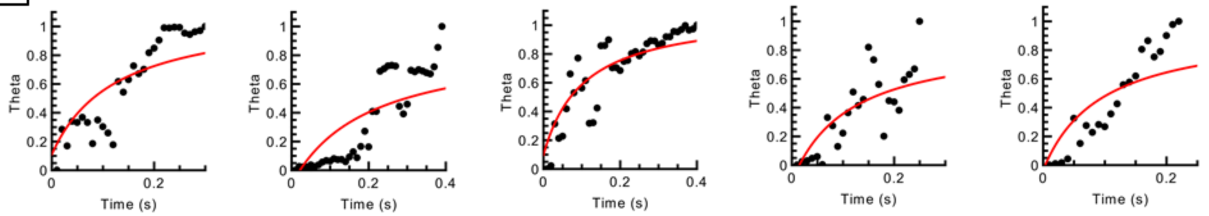
Theta
Time (s)

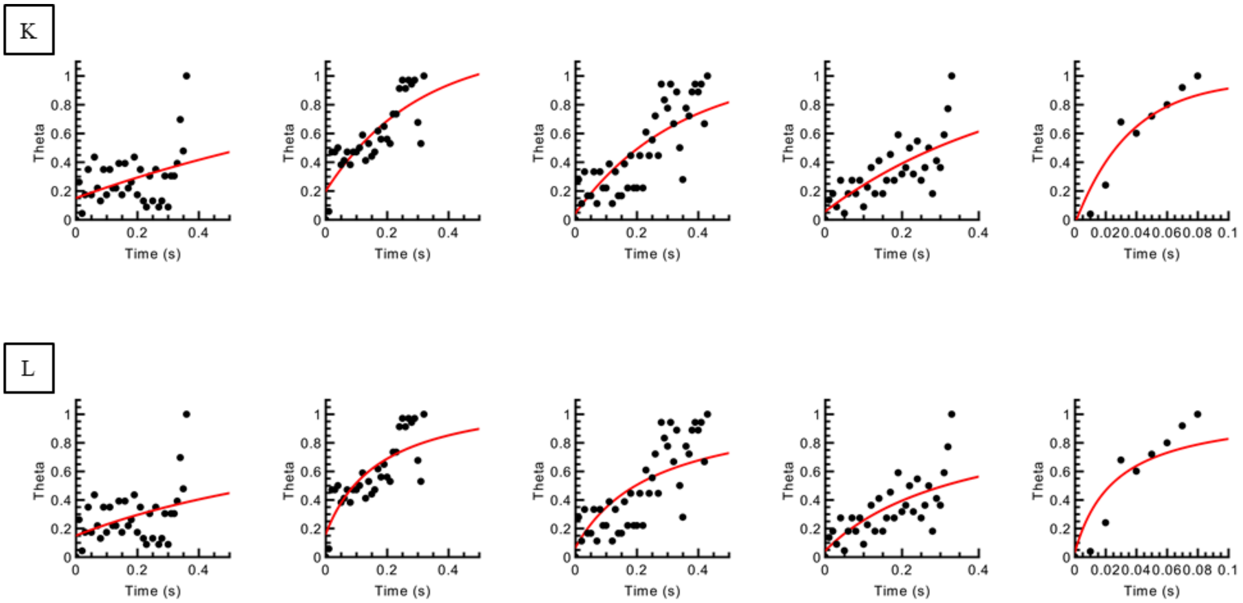

Figure S1. Experimental adsorption data of the proportion of the surface occupied (θ) by 2×10^5 ppm acetone headspace and the corresponding curve fits using the Langmuir adsorption method by the rGO sensor after cleaning (A: One-site, 400 mV; B: Two-site, 400 mV; C: One-site, 10 mV; D: Two-site, 10 mV), the GO deposited sensor (E: One-site, 400 mV; F: Two-site, 400 mV; G: One-site, 10 mV; H: Two-site, 10 mV), and the rGO sensor (I: One-site, 400 mV; J: Two-site, 400 mV; K: One-site, 10 mV; L: Two-site, 10 mV).

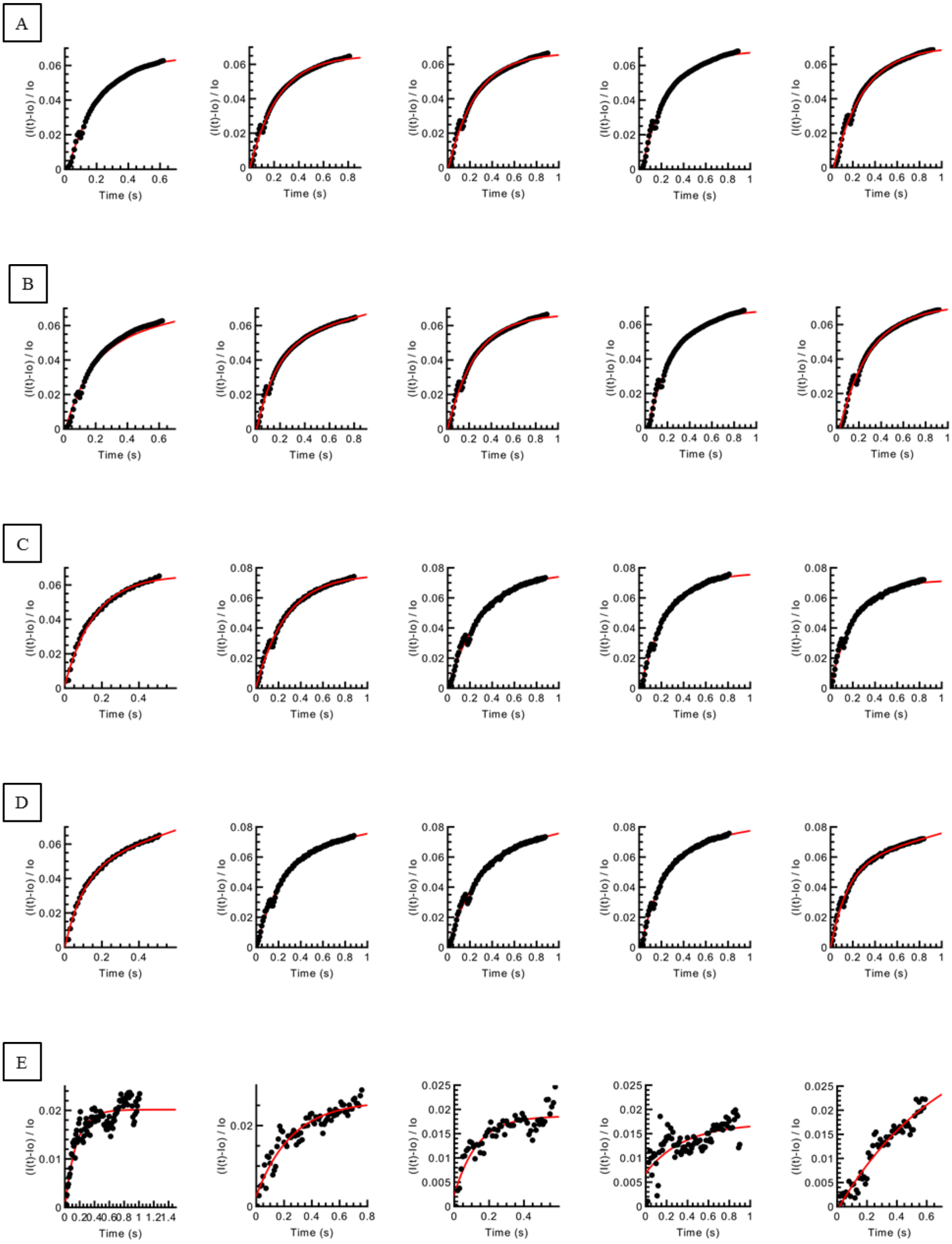
A
B
C
D
E
(I(t)-Io) / Io
Time (s)

F

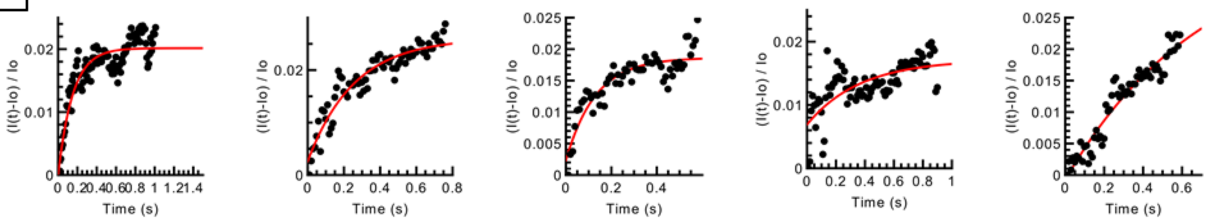
(I(t)-Io) / Io
Time (s)

G

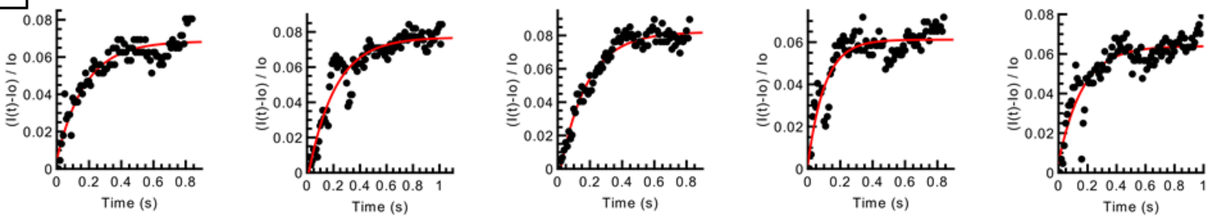
(I(t)-Io) / Io
Time (s)

H

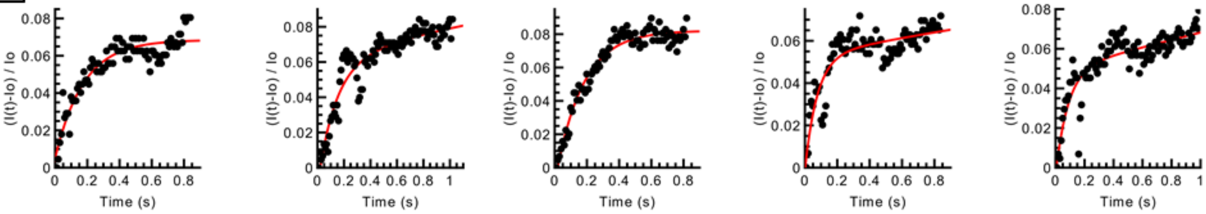
(I(t)-Io) / Io
Time (s)

I

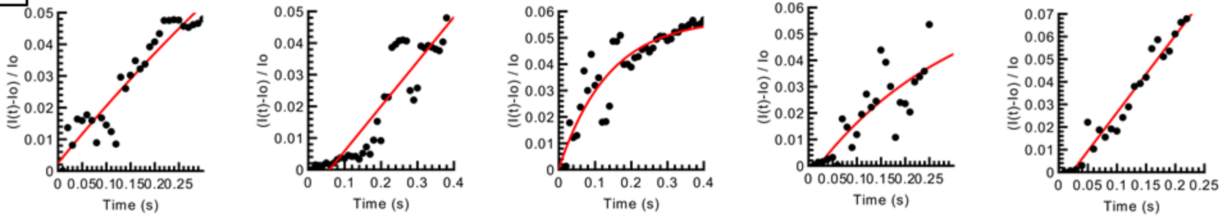
(I(t)-Io) / Io
Time (s)

J

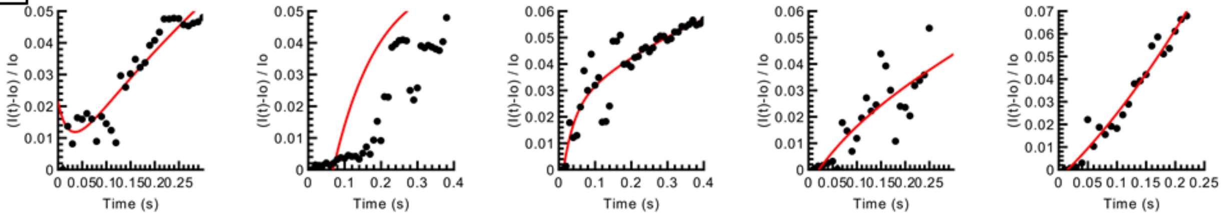
(I(t)-Io) / Io
Time (s)

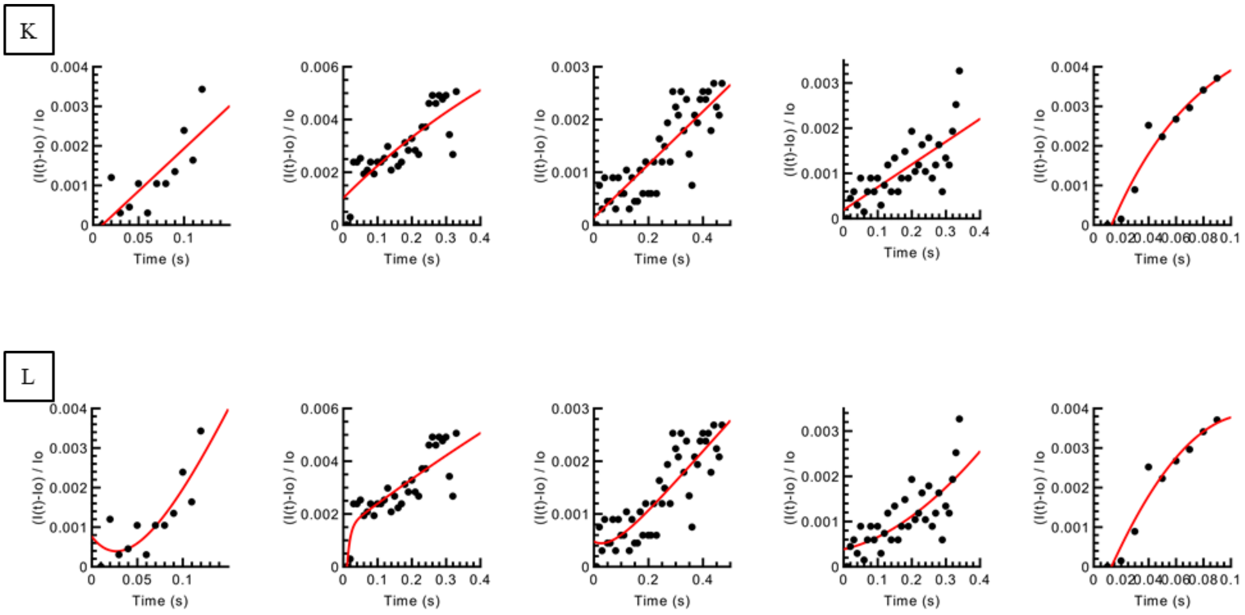

Figure S2. Experimental adsorption data of the responsiveness by 2×10^5 ppm acetone headspace and the corresponding curve fits using the single and double exponent adsorption method by the rGO sensor after cleaning (A: single exponent, 400 mV; B: double exponent, 400 mV; C: single exponent, 10 mV; D: double exponent, 10 mV), the GO deposited sensor (E: single exponent, 400 mV; F: double exponent, 400 mV; G: single exponent, 10 mV; H: double exponent, 10 mV), and the rGO sensor (I: single exponent, 400 mV; J: double exponent, 400 mV; K: single exponent, 10 mV; L: double exponent, 10 mV).

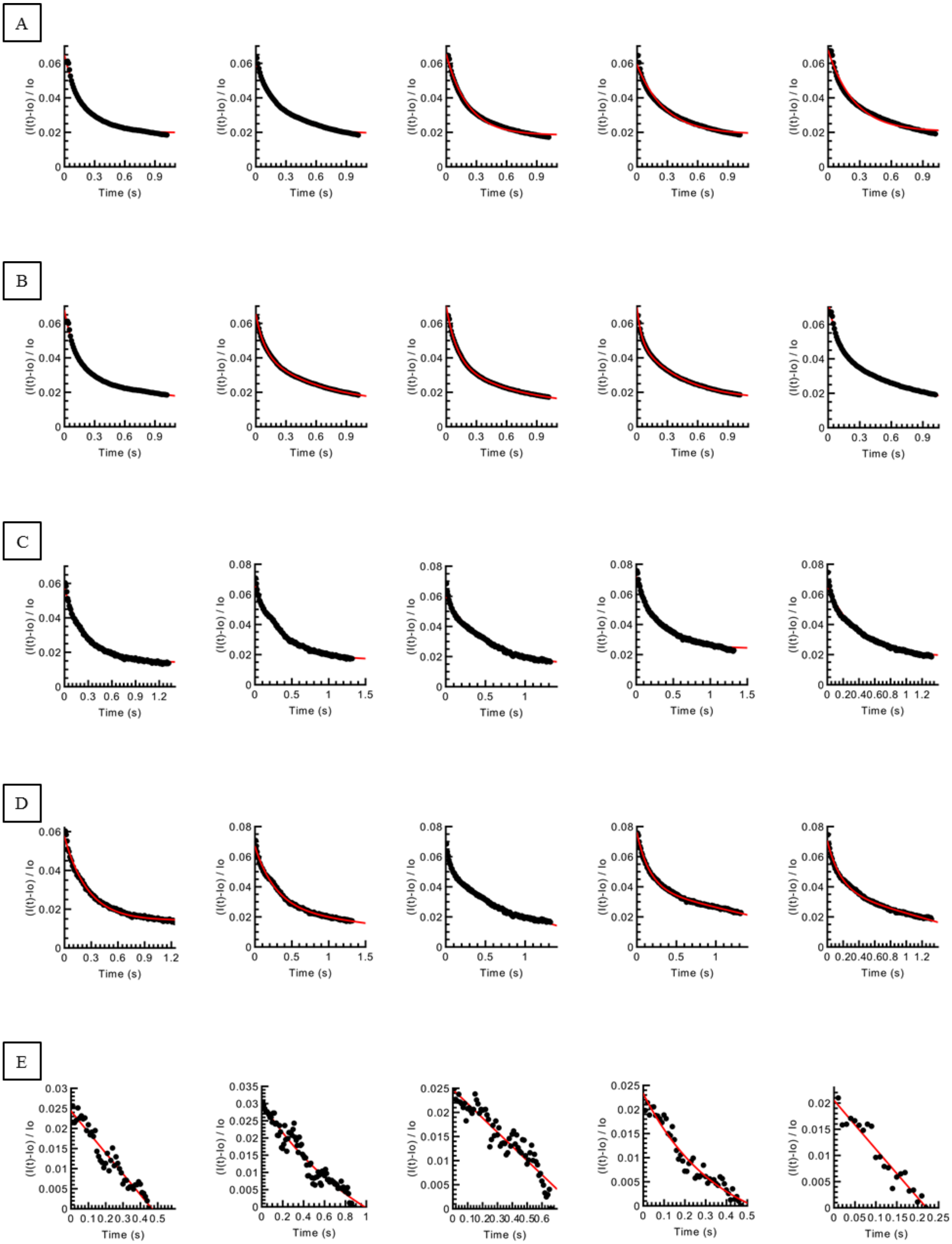
A
B
C
D
E
(I(t)-Io) / Io
Time (s)

F

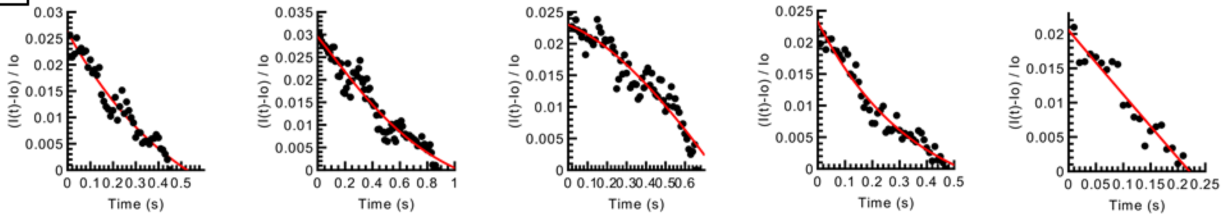
(I(t)-Io) / Io
Time (s)

G

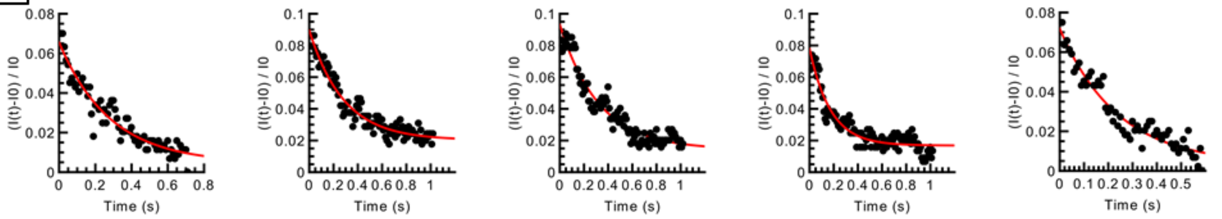
(I(t)-I0) / I0
Time (s)

H

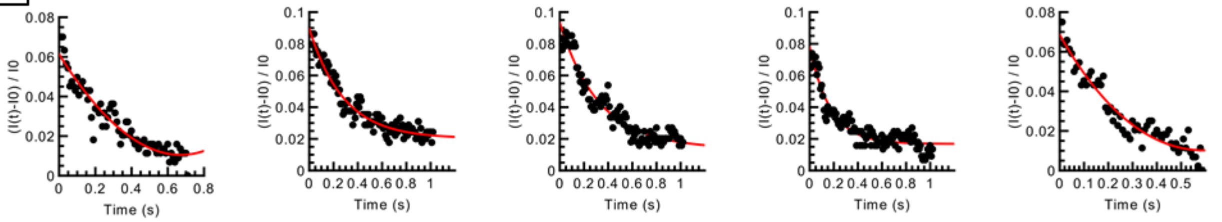
(I(t)-I0) / I0
Time (s)

I

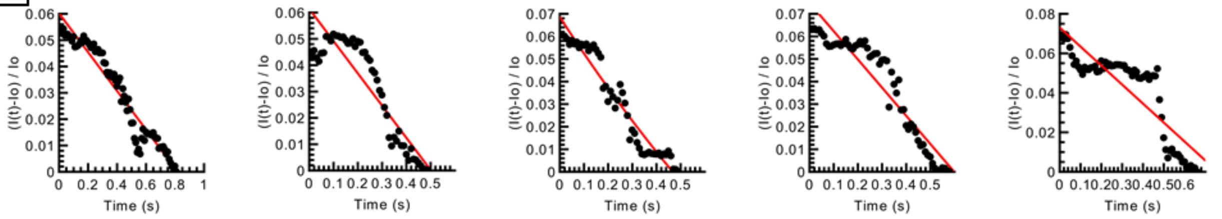
(I(t)-Io) / Io
Time (s)

J

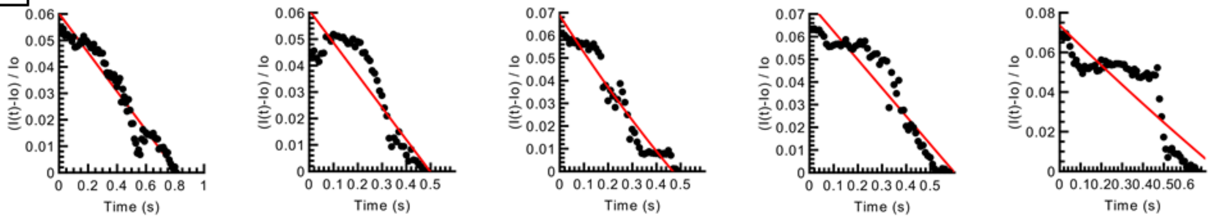
(I(t)-Io) / Io
Time (s)

K

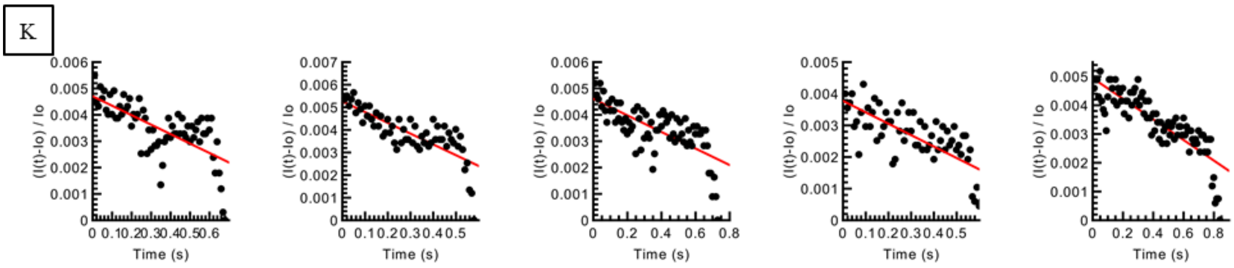

L

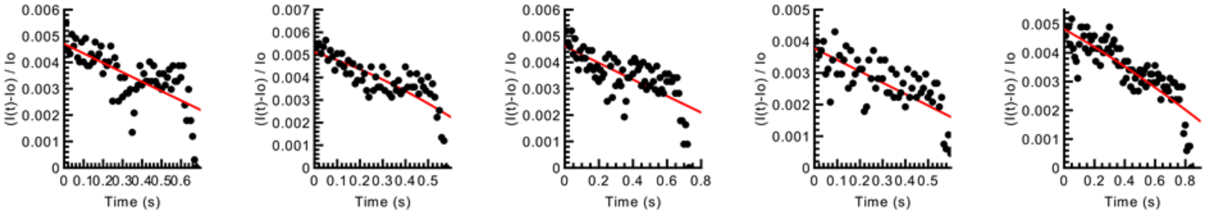

Figure S3. Experimental desorption data of the responsiveness by 2×10^5 ppm acetone headspace and the corresponding curve fits using the single and double exponent desorption method by the rGO sensor after cleaning (A: single exponent, 400 mV; B: double exponent, 400 mV; C: single exponent, 10 mV; D: double exponent, 10 mV), the GO deposited sensor (E: single exponent, 400 mV; F: double exponent, 400 mV; G: single exponent, 10 mV; H: double exponent, 10 mV), and the rGO sensor (I: single exponent, 400 mV; J: double exponent, 400 mV; K: single exponent, 10 mV; L: double exponent, 10 mV).

Table S1. Peak fit results for Lorentzian curves with amplitude, peak center, and full width at half mid-point were presented from the Raman spectra on GO, rGO, and solvent exfoliated rGO thin film.

Process		DEP	After 5 h reduction	Solvent Exfoliated
D-band	Amplitude	8442.5	32495.9	18865.5
	Center	1341.9	1350.9	1344.9
	FWHM	131.4	128.9	114.8
G-band	Amplitude	8382.5	31215.9	17481.5
	Center	1606.0	1608.0	1599.0
	FWHM	66.7	64.9	64.7
Id/Ig		1.01	1.04	1.08
2D-band	Amplitude	1273.7	3998.9	2340.8
	Center	2682.2	2692.5	2685.1
	FWHM	200.4	178.9	175.2
D+G(S3)-band	Amplitude	1646.3	6032.4	3561.2
	Center	2959.9	2930.0	2926.5
	FWHM	201.9	195.1	180.1
C-H mode stretching	Amplitude	448.7	1617.1	978.4
	Center	3186.6	3186.9	3187.1
	FWHM	50.8	75.9	71.9

Table S2. Curve fit parameter values with errors on adsorption data using the Langmuir one-site isotherm model.

Sensor	Data	Trial no.	α	β	α-error	β-error
As deposited	One-site 400 mV Adsorption	1	1.158	6.259	0.021	0.368
		2	1.218	3.067	0.022	0.204
		3	1.523	4.014	0.024	0.457
		4	1.523	4.014	0.047	2.080
		5	1.000	3.349	0.135	0.158
As deposited	One-site 10 mV Adsorption	1	1.566	3.643	0.080	0.516
		2	1.161	4.193	0.044	0.438
		3	1.161	4.193	0.044	0.043
		4	1.142	5.371	0.150	1.530
		5	1.431	4.258	0.088	0.686
rGO	One-site 400 mV Adsorption	1	1.000	6.199	0.414	1.119
		2	1.000	4.015	1.156	0.565
		3	1.000	7.244	0.077	1.839
		4	1.000	6.036	0.608	2.623
		5	1.000	8.295	0.575	0.847
rGO	One-site 10 mV Adsorption	1	1.000	0.770	0.005	0.357
		2	1.000	3.342	0.932	1.167
		3	1.000	2.946	1.981	0.574
		4	1.000	2.021	54.712	58.248
		5	1.000	27.973	0.216	7.221
Exfoliated rGO	One-site 400 mV Adsorption	1	1.000	4.230	0.007	0.220
		2	1.187	3.567	0.011	0.112
		3	1.221	2.970	0.010	0.122
		4	1.000	3.919	0.008	0.116
		5	1.000	3.681	0.008	0.113
Exfoliated rGO	One-site 10 mV Adsorption	1	1.068	6.203	0.017	0.314
		2	1.000	3.635	0.007	0.088
		3	1.000	3.384	0.008	0.105
		4	1.000	4.111	0.007	0.109
		5	1.294	3.296	0.010	0.147

Table S3. Curve fit parameter values with errors on adsorption data using the single-site and double-site exponent model.

Sensor	Data	Trial no.	Single-exponent		Double-exponent			
			τ	τ-error	τ1	τ1-error	τ2	τ2-error
As deposited	400 mV Adsorption	1	0.151	0.015	0.151	0.0164	0.271	1.24
		2	0.256	0.032	0.256	2346.06	0.257	2258.14
		3	0.131	0.022	0.131	0.01	0.237	13.31
		4	0.368	0.145	0.368	2544.82	0.357	2726.98
		5	0.706	0.254	0.706	0.000	0.966	0.27
As deposited	10 mV Adsorption	1	0.175	0.023	0.175	0.021	0.050	2598.960
		2	0.204	0.057	1.542	10.044	0.131	0.064
		3	0.169	0.037	0.165	63.548	0.169	0.912
		4	0.104	0.016	0.076	0.034	3.988	105.774
		5	0.164	0.033	0.086	0.028	1484.311	4.62E+06
rGO	400 mV Adsorption	1	1.221	2.834	0.800	70459.100	0.023	19127.500
		2	174.560	4538.180	0.124	17.924	0.047	17.124
		3	0.138	0.035	0.031	0.032	0.632	2.375
		4	0.295	0.553	200.237	913774.000	0.103	6.371
		5	600.260	11556.300	125.869	279845.000	1.519	145.942
rGO	10 mV Adsorption	1	53.03	53765.40	58.519	327170.00	0.037	0.895
		2	0.79	1.85	0.009	0.01	2.293	32.789
		3	1499.15	1946300.00	5848.846	2401530.00	0.713	0.172
		4	184.27	85654.80	16.319	25568.30	1.097	167.708
		5	0.07	0.07	0.828	20424.100	1.044	14580
Exfoliated rGO	400 mV Adsorption	1	0.205	0.005	0.157	0.037	213.257	10402600
		2	0.229	0.005	0.165	0.029	241.469	136439
		3	0.250	0.006	0.250	0.033	2.189	7.9862
		4	0.243	0.006	0.175	0.030	0.243	3153900
		5	0.271	0.007	0.203	0.077	0.373	908
Exfoliated rGO	10 mV Adsorption	1	0.161	0.005	0.1025	0.018	4.79	417635
		2	0.273	0.006	0.2259	0.037	66.35	1278000
		3	0.301	0.009	0.2472	0.044	53.80	2460130
		4	0.235	0.005	0.2074	0.014	74.28	2953190
		5	0.221	0.006	0.1500	0.009	19.33	1705870

Table S4. Curve fit parameter values with errors on desorption data using the single-site and double-site exponent model.

Sensor	Data	Trial no.	Single-exponent		Double-exponent			
			τ	τ-error	τ1	τ1-error	τ2	τ2-error
As deposited	400 mV Desorption	1	0.540	0.216	0.833	1157.83	0.678	104.7540
		2	1.083	0.329	1.065	0.19	0.425	29.2482
		3	519	17478.200	1.244	2.58	0.646	6.870
		4	0.351	0.079	0.095	0.38	1.490	804.08
		5	14.568	498.260	0.030	0.46	5.737	1461.02
As deposited	10 mV Desorption	1	0.282	0.038	0.265	0.006	5.001	5937.150
		2	0.283	0.020	0.429	0.006	0.464	763.643
		3	0.323	0.028	0.726	0.003	0.323	3.388
		4	0.174	0.015	0.119	0.002	113.016	0.034
		5	0.238	0.034	0.141	0.008	16.237	5.42E+03
rGO	400 mV Desorption	1	698.281	131258	169.691	1168770.0	159.151	1662960
		2	121.325	15703	88.191	138521.0	87.624	134981
		3	1.897	2.222	3.583	257387.0	2.435	61647
		4	249.032	40916	368.877	2081570.0	369.284	2131560
		5	644.594	338594	12.578	6063.7	1.623	271
rGO	10 mV Desorption	1	26665	62.356	74.8	7120980	1.255	2369
		2	163	453.237	48.6	284271	3.641	2394
		3	221	563.237	156.6	530520000	63.951	19584
		4	296	123.236	175.3	235625625	80.739	14074
		5	440	2565.327	172.6	114525	793.430	1436450
Exfoliated rGO	400 mV Desorption	1	0.191	0.004	0.011	0.009	1.043	0.810
		2	0.267	0.007	0.003	0.006	0.608	0.033
		3	0.220	0.005	0.005	0.005	0.667	0.083
		4	0.276	0.009	0.001	0.010	0.440	0.007
		5	0.252	0.008	0.007	0.007	0.861	0.171
Exfoliated rGO	10 mV Desorption	1	0.263	0.005	0.2627	0.005	0.113	12.325
		2	0.365	0.008	0.2949	0.060	21.536	1172.8
		3	0.471	0.016	0.2334	0.055	32.175	914.3
		4	0.314	0.008	0.1666	0.011	329.27	3826.3
		5	0.383	0.012	0.1739	0.015	1438.57	56236.4
